# Adverse effects of Janus kinase inhibitors with relevance for daily practice in dermatology

**DOI:** 10.1111/ddg.15796

**Published:** 2025-09-15

**Authors:** Anika Rajput Khokhar, Kamran Ghoreschi, Julia Huynh

**Affiliations:** ^1^ Department of Dermatology Venereology and Allergology Charité – Universitätsmedizin Berlin corporate member of Freie Universität Berlin and Humboldt‐Universität zu Berlin Berlin Germany

**Keywords:** Atopic dermatitis, Janus kinase inhibitor, Pharmacology, Psoriasis, Side effect

## Abstract

Used since 2011 in the USA and 2012 in the EU, Janus kinase inhibitors (JAKi) are gaining increasing acceptance as a treatment for dermatological diseases such as atopic dermatitis, psoriasis, psoriatic arthritis, alopecia areata, chronic hand eczema and vitiligo. Knowledge of their mechanism of action and potential side effects is necessary for a safe and effective use. Their short half‐life requires daily administration, enables good controllability and is appreciated by many patients due to the rapid onset of action and the absence of subcutaneous or intravenous injections.

Common side effects are upper respiratory tract infections as well as varicella zoster virus reactivations. Serious infections can occur in rare cases, which may take a problematic course. An increased risk of cardiovascular events has been described in certain JAKi, so alternative treatment should be preferred in patients at cardiovascular risk. In studies on rheumatoid arthritis, an increased incidence of malignancies (bronchial carcinoma, lymphoma) was observed with tofacitinib. JAKi have also been associated with more aggressive progression of epithelial skin tumors. Animal studies indicate teratogenic effects during pregnancy. Older patients and those at increased risk should only receive JAKi after careful risk‐benefit assessment. Appropriate preliminary examinations and regular laboratory monitoring are necessary to ensure safe therapy.

## MODE OF ACTION OF JANUS KINASE INHIBITORS (JAKi)

At the molecular biological level, chronic inflammatory diseases are driven by various inflammatory cascades. Proinflammatory cytokines such as tumor necrosis factor (TNF), interleukin (IL‐)6 or IL‐1 initiate the production of further factors, e.g. IL‐17A and IL‐22.^1^ These messenger molecules bind to their respective receptors on target cells, with many cytokine receptors transmitting intracellular signals via the JAK‐STAT signaling pathway. The receptor subunits dimerize, thereby initiating intracellular signal transduction. Through dimerization, Janus kinases (JAKs) are recruited to the intracellular domains of the receptor and undergo autophosphorylation, inducing a conformational change in the receptor. JAKs can transfer phosphate groups to STAT (signal transducer and activator of transcription) molecules. Once phosphorylated, STATs become activated, dimerize, and translocate into the nucleus, where they function as transcription factors to regulate target gene expression.

Over 50 different cytokines and growth factors mediate their effect via the JAK/STAT signaling pathway. Four different JAKs are found in humans: JAK1, JAK2, JAK3 and tyrosine kinase 2 (TYK2). Cytokines bind to their respective receptors, which associate with JAK or TYK molecules. The cytokine‐receptor complexes employ either two JAK2 molecules or various combinations of JAK1, JAK2, JAK3 and TYK2. A maximum of three different JAKs are activated. This activation leads to intracellular signal transduction with transcription of target genes by STAT dimers. For example, IL‐6 acts on the target cells via its receptor complex in association with JAK1, JAK2 and TYK2. Depending on the receptor complex and the associated JAK combination, specific signals are transmitted, leading to distinct effects in target cells – such as lymphocyte proliferation, myelopoiesis, or erythropoiesis.

Therapeutically, JAKi block the downstream signaling cascade by competitively binding to the ATP‐binding pocket of the kinase domain. Thus, they prevent phosphorylation and further signal transduction. JAKi therefore exert immunomodulatory, antiproliferative, and anti‐inflammatory effects, depending on the specific JAK isoform targeted.

The first generation of JAKi was not highly selective for a single Janus kinase; for example, tofacitinib exhibits high affinity not only for JAK3, but also for JAK1 and JAK2. However, further advancements have led to the development of more selective JAK inhibitors, such as JAK1‐specific agents, which enable more targeted inhibition of the cytokine receptor spectrum and offer a narrower efficacy and side effect profile compared to first‐generation JAKi.

Less selective JAKi, such as tofacitinib, represent the so‐called first generation and can be distinguished from more selective second‐generation JAKi, including upadacitinib (JAK1i), filgotinib (JAK1i), abrocitinib (JAK1i), and deucravacitinib (TYK2i).^2^ In contrast to other JAK inhibitors, deucravacitinib targets the pseudokinase domain instead of the catalytic kinase domain, resulting in selective inhibition of TYK2. Deucravacitinib thus inhibits the signaling cascade of the interleukins IL‐12 and IL‐23 as well as the type 1 interferons. JAK1–3 are not affected by deucravacitinib at therapeutic doses.^3^, ^4^

More than 50 cytokines exert their effects on target cells via the intracellular JAK‐STAT signaling pathway, following binding to their specific cytokine receptor complexes. Janus kinase inhibitors (JAKi) disrupt this pathway by inhibiting the intracellular phosphorylation of STAT molecules, thereby preventing their activation and function as transcription factors. Newer, more selective JAKi – such as upadacitinib, abrocitinib, filgotinib and deucravacitinib – enable more targeted modulation of cytokine signaling and are associated with a reduced side effect profile compared to first‐generation JAKi.

## AREAS OF APPLICATION

Currently, systemic JAKi are primarily approved for use across a range of chronic inflammatory and autoimmune diseases, including psoriasis, psoriatic arthritis (PsA), juvenile idiopathic arthritis (JIA), ankylosing spondyloarthritis (AS), ulcerative colitis (UC), atopic dermatitis (AD), and alopecia areata (Table 1).

**TABLE 1:** Overview of relevant JAKi in dermatologic daily practice.^42^, ^43^, ^44^, ^45^, ^49^, ^50^, ^51^, ^52^, ^53^, ^55^, ^56^, ^57^

	Non‐selective within the JAK family					Selective within the JAK family				
Agent	Baricitinib	Tofacitinib	Ruxolitinib		Delgocitinib	Upadacitinib	Abrocitinib	Filgotinib	Deucravacitinib	Ritlecitinib
Trade name	Olumiant^®^	Xeljanz^®^	Jakavi^®^	Opzelura^®^ 15 mg/g	Anzupgo^®^ 20 mg/g	Rinvoq^®^	Cibinqo^®^	Jyseleca^®^	Sotyktu^®^	Litfulo^®^
Authorized indications	AD, RA, AA	PsA, RA, SpA, JIA, CU	PV, Myelofibrosis (splenomegaly), GvHD	NSV	CHE, AD (USA, no EU approval, as of 01/2025)	AD, PsA, RA, SpA, CU, Crohn's disease	AD	RA, CU	Pso	AA
Method of administration	Peroral	Peroral	Peroral	Cutaneous	Cutaneous	Peroral	Peroral	Peroral	Peroral	Peroral
Dosage	AD: 4 mg 1 x/d 2 mg 1 x/d for high‐risk patients or desired dose reduction	PsA/RA/SpA: 5 mg 2 x/d	PV: 10 mg 2 x/d	NSV: lesional 2 x/d (max. 10% BSA per application)	Lesional 2 x/d (max. 5g per application)	AD, PsA: 15 mg 1 x/d For AD also 30 mg 1 x/d depending on the individual symptoms	AD: 200 mg 1 x/d 100 mg 1 x/d for high‐risk patients or desired dose reduction	RA: 200 mg 1 x/d	Pso: 6 mg 1 x/d	50 mg 1 x/d
Elimination half‐life	13 h	3 h	3 h	/	/	9–14 h	2–5 h	7 h	10 h	1,3–2,3 h
Affinity to JAK subtypes	JAK1, JAK2 > TYK2, JAK3	JAK1, JAK3 > JAK2	JAK1, JAK2		JAK1, JAK2, JAK3, TYK2	JAK1, JAK2	JAK1	JAK1	TYK2	JAK3, Kinases of the TEC family
Elimination	Ca. 70% renal	Ca. 70% hepatic	Ca. 70% renal		Ca. 50/50% renal/hepatic	Mainly hepatic	Ca. 85% renal	Ca. 85% renal	Hepatic > renal	Renal (ca. 66%) > hepatic
Dose adjustment for *systemic* application and renal/liver dysfunction *All drugs: contraindication for liver dysfunction Child Pugh C*	GFR 30–60 ml/min: 2 mg 1 x/d GFR <30 ml/min: contraindicated	GFR <30 ml/min and hemodialysis: 5 mg 1 x/d Child Pugh B: 5 mg 1 x/d	GFR <30 ml/min and hemodialysis (only on dialysis days after dialysis): 5 mg 1 x/d Child Pugh B: Dose halving		/	No GFR adjustment necessary	GFR 30–60 ml/min: Dose halving to 100 or 50 mg 1 x/d GFR <30 ml/min: 50 mg 1 x/d	GFR 15–60 ml/min: 100 mg 1 x/d GFR <15 ml/min: contraindicated	No GFR adjustment necessary	No GFR adjustment necessary

*Abbr*.: AA, alopecia areata; AD, atopic dermatitis; BSA, body surface area; CHE, chronic hand eczema (here moderate to severe); CU, ulcerative colitis; GvHD, Graft versus Host Disease; NSV, nonsegmental vitiligo; PV, polycythemia vera; PsA, psoriatic arthritis; Pso, psoriasis, RA, rheumatoid arthritis; SpA, ankylosing spondylitis; JIA, juvenile idiopathic arthritis.

To date, only ruxolitinib and fedratinib are approved JAK inhibitors used for the treatment of myeloproliferative diseases. Following EMA approval, ruxolitinib is authorized in the EU as a topical JAK inhibitor for the treatment of non‐segmental vitiligo. In the United States, it is also approved for the treatment of atopic dermatitis (AD) in patients aged 12 years and older. After the confirmation of its safety and efficacy in a phase 3 trial published in 2024, the pan‐JAK inhibitor delgocitinib was approved by the EMA for the treatment of moderate to severe chronic hand eczema.^5^

With regard to pharmacokinetics, JAKi are rapidly absorbed after oral administration, independently of food intake: agents such as tofacitinib and baricitinib reach peak plasma concentrations within 30–60 minutes, whereas newer JAKi typically reach their maximum levels within 4 hours.^2^ Depending on the specific agent, JAKi are eliminated via both hepatic and renal pathways. Therefore, when prescribing, it is essential to assess potential drug interactions and adjust dosing based on current renal function. JAKi with predominantly hepatic metabolism have a short half‐life of approximately 3 hours, necessitating twice‐daily administration. In contrast, JAKi primarily eliminated via the kidneys have longer half‐lives – typically around 9–14 hours – allowing for once‐daily dosing.^6^

Owing to their relatively rapid onset of action and short half‐life – compared to other systemic therapies – JAKi can be readily adjusted in response to acute side effects through daily oral administration. They are generally also suitable for interval therapy (off‐label), for example in patients with atopic dermatitis and seasonal symptom flares. For patients, the oral administration and short half‐life – particularly in the event of side effects such as acute infections – make JAKi an attractive treatment option.^7^ Moreover, for agents such as upadacitinib, baricitinib, and abrocitinib, two different dosage strengths are available, allowing for individual adjustment based on clinical presentation and risk profile.

By specifically modulating the immune system, JAKi have the potential to markedly improve the management of various complex inflammatory and autoimmune diseases.^8^ However, their use may also be associated with adverse effects and risks, making careful therapeutic monitoring essential – alongside the selection of an appropriate patient population and suitable JAK inhibitor.

JAKi are used in the treatment of chronic inflammatory diseases such as psoriasis, psoriatic arthritis, alopecia areata, chronic hand eczema, and atopic dermatitis, with some agents also approved for myeloproliferative disorders. Their rapid absorption and short half‐life offer advantages in terms of flexible dosing and effective management of adverse effects. Despite their clinical efficacy, the use of JAKi requires close monitoring due to potential adverse effects and associated risks.

## NON‐SPECIFIC SIDE EFFECTS

Despite their efficacy, JAKi can cause non‐specific side effects that can be challenging for patients. These include gastrointestinal symptoms such as nausea, vomiting and diarrhea, which can occur particularly at the start of treatment. Headaches, dizziness and fatigue are other possible non‐specific side effects, which usually occur in less than 10% of patients taking JAKi. Itching and skin irritation may arise when using topical JAKi.

JAKi can cause non‐specific side effects such as gastrointestinal discomfort, headaches, dizziness and fatigue, especially at the beginning of treatment. Skin irritation and itching are possible side effects of topical application.

## JAKi‐ASSOCIATED CUTANEOUS NON‐INFECTIOUS SIDE EFFECTS

**FIGURE 1:**
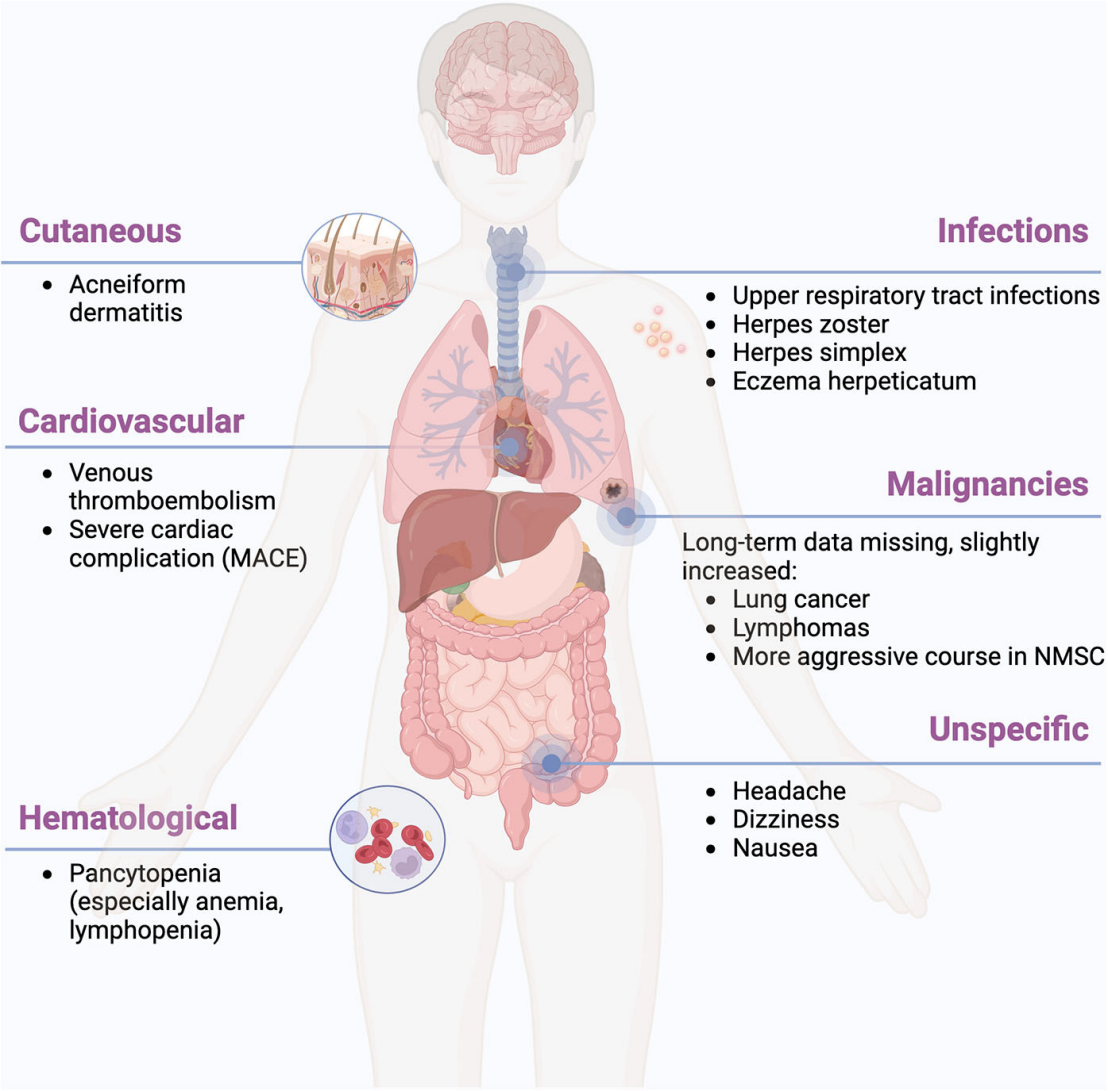
Possible adverse events associated with JAKi therapy (Figure created with biorender.com). *Abbr*.: MACE, major adverse cardiac event; NMSC, non‐melanoma skin cancer

Both systemic and topical use of JAKi is associated with an increased incidence of acneiform skin lesions (Figure 1). In a recent meta‐analysis including over 10,000 participants, 6.2% of patients treated with JAK inhibitors (JAKi) reported acne as an adverse event, compared to 1.3% in the control group.^9^ Users of abrocitinib and upadacitinib also appeared to develop acneiform lesions more frequently at higher doses (e.g., <2% with 100 mg abrocitinib vs. 4.7–5.8% with 200 mg; 1.5% with 15 mg upadacitinib vs. 3.6% with 30 mg).^10^ In most patients, the forehead and cheeks are affected. In the absence of comedones, current literature often classifies these skin changes as rosacea‐like.^11^

In previous studies on atopic dermatitis and vitiligo, topical JAK inhibitors also induced acneiform rashes at the application sites in up to 6% of cases. Additionally, pruritus at the site of application was reported by slightly fewer than 6% of users.

The recommendations for the treatment of acneiform dermatitis are based on the treatment of acne and rosacea. Usually, topical therapy with azelaic acid or benzoyl peroxide is sufficient. Systemic treatment of acneiform skin lesions with minocycline or doxycycline or even interruption of treatment with JAKi is only rarely indicated.^12^, ^13^

The use of systemic and topical JAK inhibitors has been associated with an increased risk of acneiform or rosacea‐like skin changes. These are typically managed with topical agents such as azelaic acid or benzoyl peroxide, while systemic therapy or discontinuation of JAKi is rarely required.

## RISK OF INFECTION UNDER JAK INHIBITION

The most commonly reported infections in approximately 5% of dermatological patients on JAKi include upper respiratory tract infections. The incidence rate in various clinical trials of abrocitinib and upadacitinib in patients with AD was 7–9% and 6–13% respectively, compared to 4–5% and 4–7% in the placebo group.^14^

However, infections of the upper respiratory tract or the urogenital tract are non‐specific and are found in all immunomodulatory therapies. In patients with AD receiving upadacitinib or abrocitinib, herpes simplex virus (HSV) infections or reactivations were detected more frequently (2.1–8.5% with upadacitinib vs. 0.4–1.9% with placebo; 1–2% with abrocitinib vs. 0% with placebo).^14^, ^15^, ^16^, ^17^

Reactivation of VZV as a herpes zoster infection occurred under upadacitinib in up to 5.6% of patients taking 30 mg daily and in up to 3.7% of patients taking 15 mg daily in the 52‐week follow‐up period.^15^ A herpes zoster vaccination (in Germany reimbursable from the age of 50 for certain diseases) should therefore be strongly recommended to patients before initiating therapy. The influence of JAKi on the signaling pathways of interferons and other antiviral cytokines accounts for the increased occurrence of VZV reactivations.

In patients with latent tuberculosis (TB), no cases of reactivation were reported during the 12‐ or 16‐week study periods in those treated with either abrocitinib or upadacitinib. However, patients must undergo TB screening (interferon‐gamma release assay, IGRA) prior to initiation of therapy. As expected, treatment with JAK inhibitors is contraindicated in patients with active TB. In cases of previously untreated latent TB, chemoprevention should be initiated prior to starting JAKi therapy (Table 2). According to current summaries of product characteristics, a chest X‐ray is not required as part of routine screening but may be helpful for further diagnostic clarification in the event of a positive IGRA.

**TABLE 2:** Monitoring of various JAKi in frequent dermatological use according to the summary of product characteristics.^42^, ^43^, ^44^, ^45^, ^55^, ^56^, ^58^

**Agent**	**Baricitinib **	**Tofacitinib**	**Upadacitinib**	**Abrocitinib**	**Ritlecitinib**	**Deucravacitinib**	**Ruxolitinib**	**Delgocitinib**
	Olumiant^®^	Xeljanz^®^	Rinvoq^®^	Cibinqo^®^	Litfulo^®^	Sotyktu^®^	Opzelura® 15 mg/g cream	Anzupgo^®^ 20 mg/g cream
Screening before starting therapy	CBCD, transaminases, vaccination status, TB screening, viral hepatitis screening					TB screening, vaccination status, transaminases (not for severe liver dysfunction)	Does not apply to topical ruxolitinib	Not required
Consequence of screening results							Does not apply to topical ruxolitinib	
	No start of therapy for anemia (<8 g/dl), lymphopenia (<500/mm^3^), neutropenia (<1,000/mm^3^)	No start of therapy for anemia (<9 g/dl in adults, <10 g/dl in children), lymphopenia (<750/mm^3^), neutropenia (<1,000/mm^3^, <1,200/mm^3^ in children)	No start of therapy for anemia (<8 g/dl), lymphopenia (<500/mm^3^), neutropenia (<1,000/mm^3^)	No start of therapy for anemia (<10 g/dl), thrombocytopenia (150,000/mm^3^), lymphopenia (<500/mm^3^), neutropenia (<1,200/mm^3^)	No start of therapy for thrombocytopenia (100,000/mm^3^), lymphopenia (<500/mm^3^)			
**Monitoring recommendation**								
CBCD	As part of the routine examination	After 4–8 weeks, then every 3 months	As part of the routine examination	4 weeks after the start of therapy, then as part of the routine examination		No recommendations	No recommendations	Not required
Serum lipids	12 weeks after starting therapy, then according to hyperlipidemia guideline	8 weeks after starting therapy, then according to hyperlipidemia guideline	12 weeks after starting therapy, then according to hyperlipidemia guideline	4 weeks after starting therapy, then according to hyperlipidemia guideline	No recommendations			
Transaminases	As part of the routine examination		As part of the routine examination		No recommendations			
Indications for pausing therapy	Serious infections						Does not apply to topical ruxolitinib	Pending improvement after 12 weeks of therapy
	Drug‐induced liver damage^**^ Anemia (<8 g/dl) Lymphopenia (<500/mm^3^), Tofacitinib (dose reduction/pause at 500–1,000/mm^3^, end therapy at twice <500/mm^3^) Neutropenia (<1,000/mm^3^), Tofacitinib (dose reduction/pause at <750/mm^3^, end therapy at twice <500/mm^3^) Thrombocytopenia (50,000/mm^3^)^***^				Thrombocytopenia (100,000/mm^3^), lymphopenia (<500/mm^3^)			

* Deucravacitinib not explicitly mentioned in the summary of product characteristics (caution in chronic infections).

** Not explicitly mentioned in abrocitinib's summary of product characteristics.

*** Only in abrocitinib's summary of product characteristics explicitly mentioned.

*Abbr*.: CBCD, Complete Blood Count with Differential; TB, tuberculosis

The risk of serious infections – such as those requiring hospitalization – is often considered the most critical safety outcome in clinical trials of immunotherapeutic agents. These include pneumonia, herpangina and eczema herpeticatum. The absolute event rates for serious infections were low (2 to 4 events per 100 person‐years of follow‐up) in the JAK1 trials and are comparable to those of TNF inhibitors (TNFi) – with the exception of reactivations of latent viruses such as VZV, HSV and cytomegalovirus.^14^

In the case of upadacitinib for atopic dermatitis, the risk of serious infections also appears to be dose‐dependent: 3–4 events per 100 person‐years were reported with the 30 mg daily dose, compared to 2–3 events with 15 mg. With regard to opportunistic infections, Winthrop et al. also reported a low but dose‐dependent increase in incidence in patients treated for rheumatoid arthritis (RA). Systematic analyses with long‐term data on their occurrence in dermatological indications are still lacking.^18^

Treatment with JAK inhibitors is associated with an increased risk of infections, particularly upper respiratory tract infections and reactivation of herpes viruses such as HSV and VZV. Herpes zoster vaccination is recommended prior to initiating therapy, and patients should be screened for latent tuberculosis. The risk of serious infections, including pneumonia, is comparable to that observed with TNF inhibitors and appears to be partially dose‐dependent depending on the specific JAKi.

## LIPID PROFILE AND CARDIOVASCULAR RISK

Systemic JAK inhibitors such as abrocitinib, baricitinib, tofacitinib, and filgotinib have been shown to increase both HDL and LDL levels at recommended doses. This affects up to 40% of patients and necessitates appropriate guideline‐based management of hyperlipidemia.^10^, ^19^ Whether this results in an increased long‐term cardiovascular risk remains unclear due to a lack of available data.

In the *ORAL Surveillance Study*, one of the first JAKi safety studies, an increased risk of thromboembolism was described with tofacitinib (2 × 5 mg or 2 × 10 mg) compared to TNFi. The underlying molecular pathophysiological mechanism remains still unclear. It should be noted that patients with pre‐existing cardiovascular risk were included in the *ORAL Surveillance Study*.

Further observational studies and analyses have not yet confirmed the increased cardiovascular risk associated with JAK inhibitors in rheumatologic indications within the dermatologic context, and current data on this issue remain insufficient.^14^

In a systematic review and meta‐analysis of 35 randomized clinical trials involving over 20.000 patients with dermatological disease, Ingrassia et al. detected no increased risk of major adverse cardiac events (MACE), venous thromboembolism (VTE) or all‐cause mortality with JAKi compared with placebo or an active comparator.^9^ Also among JAKi users with AD, only rare cases of MACE were reported compared to comparator drugs.^15^ Another review of over 450.000 patients from cohorts and randomized clinical trials neither identified a significant association of AD with the occurrence of VTE nor an increased risk of VTE in participants with AD receiving JAKi.^20^

Another review of the safety profile of upadacitinib in rheumatological indications (RA, PsA, SpA) observed similar rates of MACE and VTE to those of the active comparator drugs adalimumab and methotrexate.^15^ The potential risk factors described for MACE and VTE in this cohort under JAKi are already known risk factors for cardiovascular events, such as arterial hypertension, diabetes mellitus or nicotine abuse. In addition, the incidence of VTE is higher for rheumatologic indications under JAKi than for dermatologic indications.^20^, ^21^

Systemic JAKi can lead to increased HDL and LDL levels requiring guideline‐based management of hyperlipidemia. Although an increased risk of thromboembolism has been described with tofacitinib in RA, an increased cardiovascular risk from other JAKi in both rheumatological and dermatological indications has not been clearly confirmed. Observational studies and meta‐analyses have so far shown no significantly increased risk of MACE or VTE compared to placebo or comparator drugs.

## HEMATOLOGICAL CHANGES

JAK2 in particular is involved in pleiotropic signaling cascades that are crucial for the maintenance of healthy hematopoiesis, among other things. This is particularly evident in animal experiments, where JAK2‐deficient mice die *in utero* from bone marrow failure.^22^ JAK2 is activated by cytokines that induce different effects in the bone marrow: Granulopoiesis (granulocyte colony‐stimulating factor, IL‐3, granulocyte‐macrophage colony‐stimulating factor), erythropoiesis (erythropoietin), thrombopoiesis (thrombopoietin) and eosinopoiesis (IL‐5).^23^ In the pathological context of myeloproliferative disorders, such as polycythemia vera, a gain‐of‐function mutation in the JAK2 gene is often the underlying cause.^24^

For the treatment of JAK2‐mutated patients with myeloproliferative diseases, the drug inhibition of JAK2 with ruxolitinib, fedratinib and momelotinib is used. In other patient populations without JAK2 mutations, however, cytopenia can occur as a dose‐dependent side effect of first‐generation JAKi.^25^, ^26^, ^27^

Second‐generation JAK inhibitors with increased selectivity for JAK1, such as upadacitinib and filgotinib, largely avoid this side effect profile.^28^ Cytopenia has also not been observed as a complication of treatment with the TYK2 inhibitor deucravacitinib in the absence of JAK2 inhibition. Laboratory monitoring is therefore not mandatory for all JAKi. Nevertheless, it is advisable to perform a differential blood count prior to initiating therapy and periodically thereafter if clinically indicated. Medication should be discontinued in the event of anemia or a hemoglobin drop of >2 g/dl, lymphopenia, or neutropenia, in accordance with the respective product information (Table 2).^27^

Janus kinases, particularly JAK2, play a central role in hematopoiesis, and their inhibition – especially with first‐generation JAK inhibitors – can lead to dose‐dependent cytopenias. More selective second‐generation JAK inhibitors and the TYK2 inhibitor deucravacitinib are associated with a lower risk of hematologic adverse events. A differential blood count should be performed prior to initiating therapy and monitored during treatment as recommended in the prescribing information or based on clinical need.

## INFLUENCE ON MALIGNANCIES

From a mechanistic perspective, long‐term suppression of the JAK/STAT signaling pathway may increase the risk of malignancies, as type I and II interferons play a key role in the immune system's antitumor response. Conversely, the JAK/STAT pathway is also involved in cell survival and proliferation, suggesting that its effects in tumors may vary. Further studies and long‐term observational data are needed to clarify these potential risks.

In the 3‐year ORAL Surveillance study on tofacitinib in rheumatoid arthritis (RA), the risk of malignancy was slightly higher with tofacitinib compared to TNF inhibitors (1.13 vs. 0.77 events per 100 patient‐years). Notably, an increased incidence of bronchial carcinoma and lymphoma was reported in the tofacitinib group, with a hazard ratio of 1.48 compared to the TNFi‐treated RA cohort. It should be noted, however, that patients with RA already have a higher incidence of both Hodgkin and non‐Hodgkin lymphomas compared to the general population.^29^ In addition, a stratified post hoc analysis showed that the increased incidence of malignancies with tofacitinib compared to TNF inhibitors was observed only in the high‐risk group (patients >65 years or active/former long‐term smokers), but not in the low‐risk group.^30^ Non‐melanoma skin cancer (NMSC) was not investigated in the analysis.^31^

Transplant patients treated with tofacitinib showed a higher risk of lymphoproliferative malignancies. However, it should be noted that the doses of tofacitinib used in the studies were higher than the dose approved by the EMA for RA and that patients were treated with other immunosuppressants at the same time.^27^

The incidence of both melanocytic lesions and NMSC also appears to be increased with JAK inhibitor therapy. Over a 5‐year observation period, patients with rheumatoid arthritis treated with upadacitinib experienced five times more NMSC than those treated with adalimumab (0.5 vs. 0.1 events per 100 patient‐years).^32^, ^33^ Additionally, an analysis of the World Health Organization's global pharmacovigilance database identified a positive disproportionality signal – indicating a suspected drug association – for the following malignant skin tumors: squamous cell carcinoma with ruxolitinib (IC_025_ = 3.92) and tofacitinib (IC_025_ = 0.82); melanoma with ruxolitinib (IC_025_ = 0.81) and tofacitinib (IC_025_ = 0.74); and Merkel cell carcinoma with ruxolitinib (IC_025_ = 4.00), tofacitinib (IC_025_ = 1.01), and baricitinib (IC_025_ = 0.53). In addition, Merkel cell carcinoma, as a fundamentally rare disease, was particularly well represented in the reported sample and was associated with a significant disproportionality signal in all JAKi studied.^34^, ^35^ Another epidemiological study based on the *US Food and Drug Administration Adverse Event Reporting System* (FAERS) confirmed a positive association of ruxolitinib, tofacitinib and upadacitinib with malignant skin tumors. Specifically, a significant association of neuroendocrine skin tumors with ruxolitinib and squamous cell carcinoma of the skin with upadacitinib was reported.^36^ A possible risk of JAKi for photocarcinogenesis of the skin has yet to be clarified.

Although not yet systematically analyzed, in addition to the increased incidence of NMSC, more cases of patients with JAKi, especially oral ruxolitinib, with particularly aggressive epithelial tumors (mainly squamous cell carcinomas of the skin) have also been described.^37^ However, the data available to date are insufficient for a clear association, partly due to the lack of registry studies for NMSC.^38^

Long‐term inhibition of the JAK/STAT signaling pathway may increase the risk of malignancies, particularly in high‐risk groups such as older individuals or long‐term smokers. In patients with rheumatoid arthritis, tofacitinib has been associated with a higher risk of lung cancer and lymphoma compared to TNF inhibitors. Observational studies have also reported an increased incidence of malignant skin tumors in patients receiving JAK inhibitor therapy.

## PREGNANCY, LACTATION, FERTILITY

Based on animal studies, JAK inhibitors exhibit teratogenic effects during pregnancy. Genetically modified mice with functional deficiencies in JAK1 or JAK2 are non‐viable, and to date, no complete loss‐of‐function mutations in JAK1 or JAK2 have been identified in humans.^39^

It is assumed that JAKi can cross the placental barrier from the beginning of pregnancy. In animal studies, tofacitinib produced teratogenic and fetocidal effects when administered in doses above the approved human dose. Tofacitinib has been experimentally detected in the milk of lactating rats.^40^ To date, comprehensive human studies on the safety of JAK inhibitors during pregnancy or lactation are lacking; therefore, their use in these patients should be strictly avoided.^27^ Based on current data, women of childbearing age must be advised to use effective contraception during treatment with JAK inhibitors. A reliable contraceptive method should also be continued for 1 to 4 weeks after the last dose, depending on the specific agent.

Among JAK inhibitors, filgotinib has been associated with reduced fertility, impaired spermatogenesis, and histopathological changes in male reproductive organs in preclinical studies – effects that were dose‐dependent and irreversible. However, two randomized controlled trials in humans (MANTA and MANTA‐RAY) demonstrated that daily administration of 200 mg filgotinib over 13 weeks had no measurable impact on sperm parameters or sex hormone levels in men with active inflammatory bowel disease or inflammatory rheumatic diseases.^41^ The potential risk of reduced fertility or infertility with filgotinib should therefore be discussed with male patients before starting treatment.

Abrocitinib, baricitinib, upadacitinib and tofacitinib showed no effect on male fertility in animal models. The influence on fertility in humans has not been investigated.^42^, ^43^, ^44^, ^45^

According to animal studies, JAK inhibitors exhibit teratogenic effects and should therefore be avoided during pregnancy. Women of childbearing age are advised to use effective contraception during treatment and for a period after discontinuation, depending on the specific agent. While a negative impact of filgotinib on male fertility has been suggested based on preclinical data, human studies have shown no significant effects on sperm parameters at recommended doses. Other JAK inhibitors have not demonstrated adverse effects on male fertility in animal models.

## CURRENT RECOMMENDATIONS

An interdisciplinary expert panel of physicians and patient representatives recently reviewed the data of the *ORAL Surveillance Study* of tofacitinib and observational data of baricitinib. Based on the available data, it was concluded that the described risks affect all JAKi with an approved indication of a chronic inflammatory disease. Based on this review, the *Pharmacovigilance Risk Assessment Committee* (PRAC) of the EMA issued special recommendations to minimize the risk of serious side effects of JAKi in chronic inflammatory diseases.^46^ The TYK2 inhibitor deucravacitinib had not yet been approved at the time of the published PRAC recommendations and was therefore not included in the review procedure. Nevertheless, its safety profile should be considered in clinical practice, even though, as a pseudokinase inhibitor, it may have a different risk profile compared to previously approved JAK inhibitors.^46^

Due to the associated adverse effects, JAK inhibitors should be used in vulnerable patient populations only when no suitable alternative treatment is available. This includes patients aged ≥ 65 years, those with an elevated risk of serious cardiovascular events (e.g., myocardial infarction or stroke), current or long‐term former smokers, and patients with an increased risk of malignancy or venous thromboembolism (VTE). It is worth noting that the *ORAL Surveillance Study* on tofacitinib – which forms the basis for these recommendations – included only patients with at least one cardiovascular risk factor as an inclusion criterion. Notably, an increased risk of cardiovascular events was observed in the study arm receiving 10 mg twice daily.^14^, ^47^

Patients who do not belong to the above group but have risk factors for venous thromboembolism, malignancy or serious cardiovascular events should receive a reduced dose if possible.

In addition, patients receiving JAK inhibitors should be informed about the potential risk of skin cancer and undergo regular skin cancer screening. Aggressive tumor progression may occur, particularly in individuals with a history of high‐risk squamous cell carcinoma or additional risk factors such as underlying hematologic malignancies.

**FIGURE 2:**
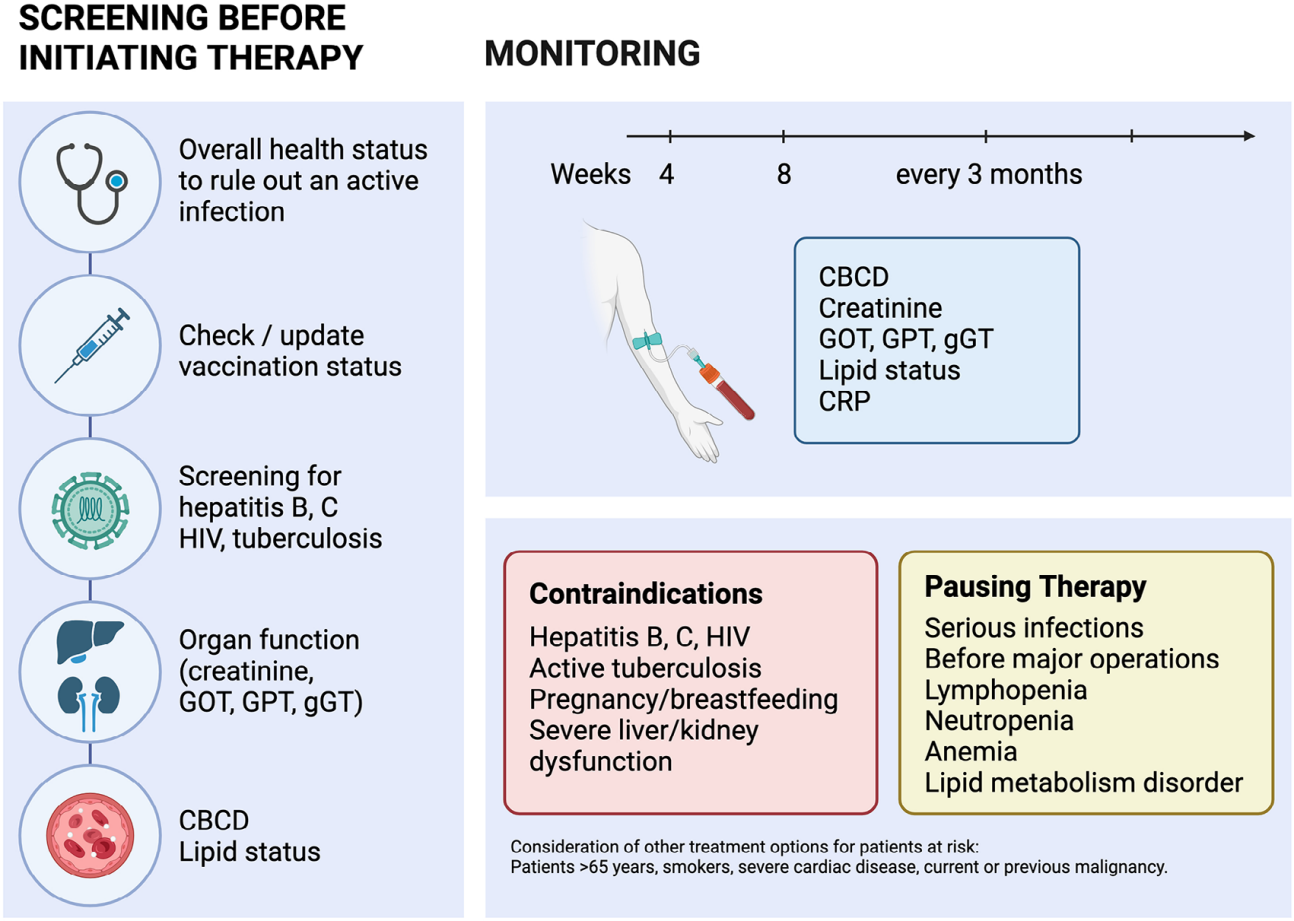
Overview of therapy preparation and monitoring under JAKi (Figure created with biorender.com).^42^, ^43^, ^44^, ^45^, ^49^, ^50^, ^51^, ^52^, ^53^ *Abbr*.: CBCD, Complete Blood Count with Differential

An indicative overview of treatment preparation and screening under JAKi is shown in Figure 2. For deucravacitinib, there are currently no manufacturer recommendations regarding laboratory monitoring during therapy. In contrast, for tofacitinib, baricitinib, ruxolitinib, upadacitinib, and abrocitinib, the recommended pre‐treatment screening and monitoring measures are summarized in Table 2, while contraindications and specific warnings from the respective product information are presented in Table 3. To minimize risks associated with the use of JAK inhibitors in dermatological patients, Wohlrab et al. developed an interdisciplinary checklist designed to pragmatically yet thoroughly identify at‐risk individuals in routine clinical practice.^48^

JAKi should only be used in high‐risk patients such as the elderly, smokers and those with cardiovascular or malignancy risks if there are no alternatives. A reduced dose is recommended for patients with risk factors for thromboembolism, malignancy or cardiovascular events. Patients should be informed about the increased risk of skin cancer and screened regularly. Specific recommendations for screening and therapy monitoring are provided in the respective summary of product characteristics.

**TABLE 3:** Contraindications and special warnings for commonly used JAKi in dermatology according to the respective summary of product characteristics.^42^, ^43^, ^44^, ^45^, ^53^, ^57^

Agent	Baricitinib	Tofacitinib	Upadacitinib	Abrocitinib	Ritlecitinib	Deucravacitinib	Ruxolitinib	Delgocitinib
Trade name	Olumiant^®^	Xeljanz^®^	Rinvoq^®^	Cibinqo^®^	Litfulo^®^	Sotyktu^®^	Opzelura^®^ 15 mg/g cream	Anzupgo^®^ 20 mg/g cream
Absolute contraindications	Pregnancy/lactation period Hypersensitivity to active substance/components							Hypersensitivity to active substance/components
	Active TB/serious infection Severe liver dysfunction					Not recommended for patients with severe liver dysfunction	Not applicable for ruxolitinib	
Special warnings	Initiate therapy for untreated latent TB No live vaccinations under therapy Risk factors for malignancies, only if no treatment alternatives available^*^ Use with caution in patients ≥ 65 years^**^ Use only after risk‐benefit assessment: Atherosclerosis, cardiovascular risk factors (smokers/former long‐term smokers)^**^ Use only after risk‐benefit assessment: in case of chronic/recurrent infections/exposure to TB^***^ origin from/travel to areas with endemic TB/mycoses^*^, ^***^				Initiate therapy for untreated latent TB Avoid live vaccinations during therapy Caution in patients with an increased risk of thromboembolism	Initiate therapy for latent TB No live vaccinations under therapy Use with caution in patients ≥ 75 years Caution in patients with chronic infections	Not for use on the eye, oral administration, intravaginal use	Not for use on eyes, mouth and other mucous membranes As a precaution, do not use during pregnancy When used during breastfeeding, contact with the nipples should be avoided
Precautions during therapy	Contraception for women of childbearing age during therapy and up to 4 weeks after the end of therapy Regular skin cancer screening Abrocitinib: Avoid combination with strong immunosuppressants/not investigated					No recommendations	Contraception, skin cancer screening	Regular examination of the application site
	/	Attention to CYP3A4 interactions	Attention to CYP3A4 interactions	Attention to CYP2C9/CYP2C19 interactions	Attention to CYP3A4, CYP1A2 interactions			

*Abbr*.: TB, tuberculosis

* Not explicitly mentioned in deucravacitinib's summary of product characteristics/lack of data.

** Does not apply to baricitinib

*** Not explicitly mentioned in summary of product characteristics of tofacitinib and baricitinib

## CONCLUSIONS

JAK inhibitors are small molecules that have already demonstrated considerable success in the treatment of various inflammatory dermatological diseases, and both the number of approved indications and the population of treated patients are expected to increase significantly in the future.^54^

Given the non‐negligible safety profile of JAK inhibitors, transparent patient education, careful preparation prior to treatment initiation, and adequate monitoring of adverse effects throughout the course of therapy are essential. The assessment of recommended risk factors and vaccination status should also be documented in writing. In general, vaccination status – not only for varicella‐zoster virus (VZV) – should be reviewed and updated prior to initiating treatment. A comprehensive assessment of long‐term safety will only be possible once robust long‐term data are available. At present, potential risks associated with prolonged use of JAK inhibitors in both adults and children remain unclear.

## CONFLICT OF INTEREST STATEMENT

A.R.K. states that she has received honoraria for lectures from Recordati Rare Diseases Germany in the last five years. J.H. states that she has received fees for consultancy and/or lectures and/or sponsorship from MSD and Kyowa Kirin in the last five years. K.G. states that he has received fees for consultancy and/or lectures and/or sponsorship for scientific projects and/or has acted as an investigator in clinical trials from or for the following companies relevant to this work in the last five years: AbbVie, Bristol Myers Squibb, LEO Pharma, Lilly, Pfizer, Incyte.

## CME Questions/Lernerfolgskontrolle

1. Welche Aussage ist richtig?
  - JAKi hemmen selektiver spezifische Zytokine als Biologika.
  - JAKi wirken, je nach Zieleinheit der JAK, immunmodulierend, antiproliferativ und entzündungshemmend.
  - Upadacitinib ist ein JAKi der ersten Generation.
  - Upadacitinib ist kein selektiver JAKi.
  - Tofacitinib ist zur Behandlung der Alopecia areata zugelassen.
2. Welche unerwünschte Wirkung/Erkrankung tritt am häufigsten als Nebenwirkung unter JAKi auf?
  - Myelosuppression
  - Herz‐Kreislauf‐Ereignisse
  - Malignome
  - Infektionen
  - Hepatotoxizität
3. Welche Aussage ist **falsch**?
  - JAKi können zum Anstieg der LDL‐Werte führen.
  - Unter Deucravacitinib sind Zytopenien nicht als Komplikation beschrieben.
  - Insbesondere JAK1 ist für die Aufrechterhaltung der Hämatopoese essenziell.
  - Ruxolitinib wird zur Behandlung myeloproliferativer Erkrankungen eingesetzt.
  - Unter oralen JAKi ist eine Kontrolle des Differenzialblutbildes im Verlauf empfohlen.
4. Welcher JAKi ist in der EU zur topischen Therapie des mittelschweren chronischen Handekzems des Erwachsenen, bei denen topische Kortikosteroide nicht ausreichen oder nicht geeignet sind, zugelassen?
  - Tofacitinib
  - Abrocitinib
  - Delgocitinib
  - Upadacitinib
  - Ruxolitinib
5. Welche Aussage ist richtig?
  - JAKi mit hepatischer Metabolisierung werden schneller ausgeschieden als JAKi mit renaler Metabolisierung.
  - Ruxolitinib ist in der EU der einzige zugelassene topische JAKi.
  - Die Sicherheit von JAKi in Schwangerschaft und Stillzeit ist durch Studien belegt.
  - JAKi sind nicht für die Therapie der Colitis ulcerosa zugelassen.
  - Akneiforme Hautveränderungen sind als dermatologische Nebenwirkungen unter JAKi nicht beschrieben.
6. Welcher JAKi kann laut Fachinformation in der Stillzeit angewandt werden?
  - Baricitinib, peroral
  - Abrocitinib, peroral
  - Deucravacitinib, peroral
  - Ruxolitinib, topisch
  - Delgocitinib, topisch
7. Welcher Parameter gehört **nicht** zum Sicherheitsmonitoring im Verlauf unter JAKi?
  - Kreatinin
  - γGT
  - Gesamtcholesterin
  - HIV‐Test
  - Anamnese zu klinischen Infektzeichen
8. Sie klären Ihren Patienten vor Therapieeinleitung über das erhöhte Risiko von Herpes zoster unter Upadacitinib auf. Wie viele Patienten unter Upadacitinib sind innerhalb eines Jahres davon betroffen?
  - Etwa jeder Zweite
  - Etwa jeder Dritte
  - Etwa jeder Vierte
  - Etwa jeder Zehnte
  - Etwa jeder Zwanzigste
9. Sie führen im Rahmen des Therapiemonitoring eine Laborkontrolle bei einem ihrer Patienten unter Upadacitinib vor. Bei welchem Parameter würden Sie die Therapie pausieren?
  - Hämoglobin 9,2 g/dl
  - Thrombozyten 193/nl
  - Neutrophile 1050/µl
  - Lymphozyten 442/µl
  - LDL‐Cholesterin 154 mg/dl
10. Sie beobachten in der Laborkontrolle Ihres Patienten mit Alopecia areata unter Baricitinib 4 mg 1 x täglich eine Anämie mit einem Hämoglobin von 7,9 g/dl. Sie haben kürzlich eine suffiziente Kontrolle der Krankheitsaktivität erreicht. Welche Option zur weiteren Behandlung wählen Sie?
  - Therapiefortsetzung
  - Dosissteigerung
  - Therapieunterbrechung
  - Dosisreduktion
  - Zusätzliche Eisensubstitution

Liebe Leserinnen und Leser, der Einsendeschluss an die DDA fur diese Ausgabe ist der 30. November 2025.

Die richtige Lösung zum Thema Unerwünschte Wirkungen von Januskinase‐Inhibitoren mit Relevanz für den dermatologischen Klinik‐ und Praxisalltag in Heft 06/2025 ist: 1a, 2c, 3e, 4d, 5a, 6a, 7c, 8c, 9c, 10e

Bitte verwenden Sie fur Ihre Einsendung das aktuelle Formblatt auf der folgenden Seite oder aber geben Sie Ihre Lösung online unter http://jddg.akademie-dda.de ein.

## References

[1] Sabat R , Wolk K , Loyal L , et al. T cell pathology in skin inflammation. Semin Immunopathol. 2019;41(3):359‐377. 31028434 10.1007/s00281-019-00742-7 PMC6505509

[2] Eichner A , Wohlrab J . Pharmacology of inhibitors of Janus kinases – Part 1: Pharmacokinetics. J Dtsch Dermatol Ges. 2022;20(11):1485‐1499. 10.1111/ddg.14921 36321475

[3] Klein B , Treudler R , Simon JC . JAK‐inhibitors in dermatology – small molecules, big impact? Overview of the mechanism of action, previous study results and potential adverse effects. J Dtsch Dermatol Ges. 2022;20(1):19‐24. 10.1111/ddg.14668 34962052

[4] Stolte KN , Mesas‐Fernández A , Meier K , et al. TYK2 inhibition with deucravacitinib ameliorates erosive oral lichen planus. Exp Dermatol. 2024;33(4):e15080. 38628035 10.1111/exd.15080

[5] Bissonnette R , Warren RB , Pinter A , et al. Efficacy and safety of delgocitinib cream in adults with moderate to severe chronic hand eczema (DELTA 1 and DELTA 2): results from multicentre, randomised, controlled, double‐blind, phase 3 trials. Lancet. 2024;404(10451):461‐473. 39033766 10.1016/S0140-6736(24)01027-4

[6] Traidl S , Freimooser S , Werfel T . Janus kinase inhibitors for the therapy of atopic dermatitis. Allergol Select. 2021;5:293‐304. 34532638 10.5414/ALX02272E PMC8439108

[7] de Souza S , Williams R , Nikiphorou E . Clinician and patient views on janus kinase inhibitors in the treatment of inflammatory arthritis: a mixed methods study. BMC Rheumatol. 2024;8(1):1. 38229170 10.1186/s41927-023-00370-7 PMC10792861

[8] Ghoreschi K , Jesson MI , Li X , et al. Modulation of innate and adaptive immune responses by tofacitinib (CP‐690,550). J Immunol. 2011;186(7):4234‐4243. 21383241 10.4049/jimmunol.1003668 PMC3108067

[9] Martinez J , Manjaly C , Manjaly P , et al. Janus Kinase Inhibitors and Adverse Events of Acne: A Systematic Review and Meta‐Analysis. JAMA Dermatol. 2023;159(12):1339‐1345. 37851459 10.1001/jamadermatol.2023.3830 PMC10585588

[10] Samuel C , Cornman H , Kambala A , Kwatra SG . A Review on the Safety of Using JAK Inhibitors in Dermatology: Clinical and Laboratory Monitoring. Dermatol Ther (Heidelb). 2023;13(3):729‐749. 36790724 10.1007/s13555-023-00892-5 PMC9930707

[11] Hölzle I , Volc S , Wehner‐Caroli J , Schaller M . Upadacitinib induced erythema, papules and pustules ‐ Is it really acne or is it rosacea? J Dtsch Dermatol Ges. 2024;22(9):1269‐1271. 38988139 10.1111/ddg.15452

[12] Ballanger F , Auffret N , Leccia MT , et al. Acneiform Lesions but not Acne after Treatment with Janus Kinase Inhibitors: Diagnosis and Management of Janus Kinase‐acne. Acta Derm Venereol. 2023;103:adv11657. 37345975 10.2340/actadv.v103.11657 PMC10296536

[13] Correia C , Antunes J , Filipe P . Management of acne induced by JAK inhibitors. Dermatol Ther. 2022;35(9):e15688. 35789037 10.1111/dth.15688

[14] Winthrop KL , Cohen SB . Oral surveillance and JAK inhibitor safety: the theory of relativity. Nat Rev Rheumatol. 2022;18(5):301‐304. 35318462 10.1038/s41584-022-00767-7 PMC8939241

[15] Guttman‐Yassky E , Teixeira HD , Simpson EL , et al. Once‐daily upadacitinib versus placebo in adolescents and adults with moderate‐to‐severe atopic dermatitis (Measure Up 1 and Measure Up 2): results from two replicate double‐blind, randomised controlled phase 3 trials. Lancet. 2021; 397(10290):2151‐2168. 34023008 10.1016/S0140-6736(21)00588-2

[16] Reich K , Teixeira HD , de Bruin‐Weller M , et al. Safety and efficacy of upadacitinib in combination with topical corticosteroids in adolescents and adults with moderate‐to‐severe atopic dermatitis (AD Up): results from a randomised, double‐blind, placebo‐controlled, phase 3 trial. Lancet. 2021;397(10290):2169‐2181. 34023009 10.1016/S0140-6736(21)00589-4

[17] Simpson EL , Sinclair R , Forman S , et al. Efficacy and safety of abrocitinib in adults and adolescents with moderate‐to‐severe atopic dermatitis (JADE MONO‐1): a multicentre, double‐blind, randomised, placebo‐controlled, phase 3 trial. Lancet. 2020;396(10246):255‐266. 32711801 10.1016/S0140-6736(20)30732-7

[18] Winthrop K , Isaacs J , Calabrese L , et al. Opportunistic infections associated with Janus kinase inhibitor treatment for rheumatoid arthritis: A structured literature review. Semin Arthritis Rheum. 2023;58:152120. 36347212 10.1016/j.semarthrit.2022.152120

[19] Li N , Gou ZP , Du SQ , et al. Effect of JAK inhibitors on high‐ and low‐density lipoprotein in patients with rheumatoid arthritis: a systematic review and network meta‐analysis. Clin Rheumatol 2022;41(3):677‐688. 34993729 10.1007/s10067-021-06003-z

[20] Chen TL , Lee LL , Huang HK , et al. Association of Risk of Incident Venous Thromboembolism With Atopic Dermatitis and Treatment With Janus Kinase Inhibitors: A Systematic Review and Meta‐analysis. JAMA Dermatol. 2022;158(11):1254‐1261. 36001310 10.1001/jamadermatol.2022.3516 PMC9403856

[21] Molander V , Bower H , Frisell T , et al. Venous thromboembolism with JAK inhibitors and other immune‐modulatory drugs: a Swedish comparative safety study among patients with rheumatoid arthritis. Ann Rheum Dis. 2023;82(2):189‐197. 36150749 10.1136/ard-2022-223050 PMC9887398

[22] Akada H , Akada S , Hutchison RE , et al. Critical role of Jak2 in the maintenance and function of adult hematopoietic stem cells. Stem Cells 2014;32(7):1878‐1889. 24677703 10.1002/stem.1711 PMC4063883

[23] Rane SG , Reddy EP . Janus kinases: components of multiple signaling pathways. Oncogene. 2000;19(49):5662‐5679. 11114747 10.1038/sj.onc.1203925

[24] Levine RL , Wadleigh M , Cools J , et al. Activating mutation in the tyrosine kinase JAK2 in polycythemia vera, essential thrombocythemia, and myeloid metaplasia with myelofibrosis. Cancer Cell. 2005;7(4):387‐397. 15837627 10.1016/j.ccr.2005.03.023

[25] Kay J , Harigai M , Rancourt J , et al. Changes in selected haematological parameters associated with JAK1/JAK2 inhibition observed in patients with rheumatoid arthritis treated with baricitinib. RMD Open 2020;6(3):e001370. 33028675 10.1136/rmdopen-2020-001370 PMC7722368

[26] Cervantes F , Ross DM , Radinoff A , et al. Efficacy and safety of a novel dosing strategy for ruxolitinib in the treatment of patients with myelofibrosis and anemia: the REALISE phase 2 study. Leukemia. 2021;35(12):3455‐3465. 34017073 10.1038/s41375-021-01261-x PMC8632662

[27] Bonelli M , Kerschbaumer A , Kastrati K , et al. Selectivity, efficacy and safety of JAKinibs: new evidence for a still evolving story. Ann Rheum Dis. 2024;83(2):139‐160. 37923366 10.1136/ard-2023-223850 PMC10850682

[28] Traves PG , Murray B , Campigotto F , et al. JAK selectivity and the implications for clinical inhibition of pharmacodynamic cytokine signalling by filgotinib, upadacitinib, tofacitinib and baricitinib. Ann Rheum Dis. 2021;80(7):865‐875. 33741556 10.1136/annrheumdis-2020-219012 PMC8237188

[29] Klein A , Polliack A , Gafter‐Gvili A . Rheumatoid arthritis and lymphoma: Incidence, pathogenesis, biology, and outcome. Hematol Oncol. 2018;36(5):733‐739. 29862535 10.1002/hon.2525

[30] Kristensen LE , Danese S , Yndestad A , et al. Identification of two tofacitinib subpopulations with different relative risk versus TNF inhibitors: an analysis of the open label, randomised controlled study ORAL Surveillance. Ann Rheum Dis. 2023;82(7):901‐910. 36931693 10.1136/ard-2022-223715 PMC10314011

[31] Kalanovic D KD . XELJANZ (TOFACITINIB): Erste Ergebnisse einer klinischen Studie deuten auf ein erhöhtes Risiko für schwerwiegende unerwünschte kardiovaskuläre Ereignisse und maligne Erkrankungen (ohne NMSC) bei der Verwendung von Tofacitinib im Vergleich zu TNF‐alpha‐Inhibitoren. In: Bundesinstitut für Arzneimittel und Medizinprodukte (BfArM) P, Europäischen Arzneimittel‐Agentur (EMA), editor. Berlin; 2021.

[32] Fleischmann R , Swierkot J , Penn SK , et al. Long‐term safety and efficacy of upadacitinib versus adalimumab in patients with rheumatoid arthritis: 5‐year data from the phase 3, randomised SELECT‐COMPARE study. RMD Open. 2024;10(2):e004007. 38806190 10.1136/rmdopen-2023-004007 PMC11138271

[33] Burmester GR , Cohen SB , Winthrop KL , et al. Safety profile of upadacitinib over 15 000 patient‐years across rheumatoid arthritis, psoriatic arthritis, ankylosing spondylitis and atopic dermatitis. RMD Open. 2023;9(1):e002735. 36754548 10.1136/rmdopen-2022-002735 PMC9923346

[34] Jalles C , Lepelley M , Mouret S , et al. Skin cancers under Janus kinase inhibitors: A World Health Organization drug safety database analysis. Therapie. 2022;77(6):649‐656. 35710462 10.1016/j.therap.2022.04.005

[35] Kreher MA , Konda S , Noland MMB , et al. Risk of melanoma and nonmelanoma skin cancer with immunosuppressants, part II: Methotrexate, alkylating agents, biologics, and small molecule inhibitors. J Am Acad Dermatol. 2023;88(3):534‐542. 36460256 10.1016/j.jaad.2022.11.043

[36] Liu T , Gao R , Li L , et al. Analysis of the association between Janus kinase inhibitors and malignant skin tumors using the Food and Drug Administration Adverse Event Reporting System. Int J Clin Pharm. 2023;45(6):1483‐1491. 37606843 10.1007/s11096-023-01634-5

[37] Lee GH , Guzman AK , Divito SJ , et al. Cutaneous Squamous‐Cell Carcinoma after Treatment with Ruxolitinib or Belumosudil. N Engl J Med. 2023;389(2):188‐190. 37437151 10.1056/NEJMc2304157

[38] Greif CS , Srivastava D , Nijhawan RI . Janus Kinase Inhibitors and Non‐Melanoma Skin Cancer. Curr Treat Options Oncol. 2021;22(2):11. 33423161 10.1007/s11864-020-00815-y

[39] Ott N , Faletti L , Heeg M , et al. JAKs and STATs from a Clinical Perspective: Loss‐of‐Function Mutations, Gain‐of‐Function Mutations, and Their Multidimensional Consequences. J Clin Immunol. 2023;43(6):1326‐1359. 37140667 10.1007/s10875-023-01483-x PMC10354173

[40] Julsgaard M , Mahadevan U , Vestergaard T , et al. Tofacitinib concentrations in plasma and breastmilk of a lactating woman with ulcerative colitis. Lancet Gastroenterol Hepatol. 2023;8(8):695‐697. 37269871 10.1016/S2468-1253(23)00158-9

[41] Reinisch W , Hellstrom W , Dolhain R , et al. Effects of filgotinib on semen parameters and sex hormones in male patients with inflammatory diseases: results from the phase 2, randomised, double‐blind, placebo‐controlled MANTA and MANTA‐RAy studies. Ann Rheum Dis. 2023;82(8):1049‐1058. 37137672 10.1136/ard-2023-224017 PMC10359529

[42] Rinvoq: EPAR – Product information. 2019. Available from: https://ec.europa.eu/health/documents/community‐register/2021/20210122150644/anx_150644_de.pdf (Last accessed January 3, 2025).

[43] Fachinformation Olumiant®. 2023. Available from: https://www.lilly.com/at/assets/pdf/fachinformation/at_olumiant_fachinformation.pdf (Last accessed January 3, 2025).

[44] Fachinformation XELJANZ® 5/10mg Filmtabletten. 2023. Available from: https://figi.pfizer.de/sites/default/files/FI‐16033.pdf (Last accessed January 3, 2025).

[45] Fachinformation Cibinqo® 50/100/200mg Filmtabletten. 2023. Available from: https://figi.pfizer.de/sites/default/files/FI‐23560.pdf (Last accessed January 3, 2025).

[46] Agency EM . Janus Kinase inhibitors (JAKi) Article‐20 procedure – EMA confirms measures to minimise risk of serious side effects with Janus kinase inhibitors for chronic inflammatory disorders. 2023.

[47] Ytterberg SR , Bhatt DL , Mikuls TR , et al. Cardiovascular and Cancer Risk with Tofacitinib in Rheumatoid Arthritis. N Engl J Med. 2022;386(4):316‐326. 35081280 10.1056/NEJMoa2109927

[48] Wohlrab J , Kegel T , Große R , Eichner A . Handlungsempfehlungen zur Risikominimierung beim Einsatz von Januskinase‐Inhibitoren zur Therapie chronisch‐entzündlicher Hauterkrankungen. J Dtsch Dermatol Ges. 2023;21(8):845‐852. 10.1111/ddg.15136_g 37574686

[49] Fachinformation Jakavi® Tabletten. 2022. Available from: https://www.fachinfo.de/pdf/014060 (Last accessed January 3, 2025).

[50] Jakavi: EPAR ‐ Product Information. 2012. Available from: https://ec.europa.eu/health/documents/community‐register/2017/20171201139377/anx_139377_de.pdf (Last accessed January 3, 2025).

[51] Kusuri‐no‐Shiori Delgocitinib. 2022. https://www.rad‐ar.or.jp/siori/english/search/result?n=43533 (Last accessed March 9, 2024).

[52] Jyseleca: EPAR ‐ Product information. 2020. Available from: https://ec.europa.eu/health/documents/community‐register/2020/20200924148982/anx_148982_de.pdf (Last accessed January 3, 2025).

[53] Fachinformation SOTYKTU® 6 mg Filmtabletten. 2023. Available from: https://fi.b‐ms.de/SOTYKTU (Last accessed January 3, 2025).

[54] Solimani F , Meier K , Ghoreschi K . Emerging Topical and Systemic JAK Inhibitors in Dermatology. Front Immunol. 2019;10:2847. 31849996 10.3389/fimmu.2019.02847 PMC6901833

[55] Sotyktu: EPAR – Product information. 2023. Available from: https://www.ema.europa.eu/de/documents/product‐information/sotyktu‐epar‐product‐information_de.pdf (Last accessed January 3, 2025).

[56] Fachinformation Litfulo® 50 mg Hartkapseln. 2024. Available from: https://labeling.pfizer.com/ShowLabeling.aspx?id=19915 Last (Last accessed January 3, 2025).

[57] Anzupgo: EPAR – Product information. 2024. Available from: https://www.ema.europa.eu/de/documents/product‐information/anzupgo‐epar‐product‐information_de.pdf (Last accessed January 3, 2025).

[58] Opzelura: EPAR – Product information. 2023. Available from: https://www.ema.europa.eu/de/documents/product‐information/opzelura‐epar‐product‐information_de.pdf (Last accessed January 3, 2025).

